# Methylamphetamine toxicity and its involvement in death: A retrospective observational study of deaths reported to the Victorian Coroner, Australia

**DOI:** 10.1007/s12024-023-00724-0

**Published:** 2023-10-04

**Authors:** Dylan Mantinieks, Melanie Archer, Jennifer Schumann, Olaf H. Drummer, Dimitri Gerostamoulos

**Affiliations:** 1 Department of Forensic Medicine, School of Public Health and Preventive Medicine, Monash University, 65 Kavanagh Street, Southbank 3006, Victoria, Australia; 2 Victorian Institute of Forensic Medicine, 65 Kavanagh Street, Southbank 3006, Victoria, Australia

**Keywords:** Central Nervous System Stimulants, Amphetamines, Methylamphetamine, Forensic Toxicology, Cause of Death, Sudden Cardiac Death, Forensic Pathology

## Abstract

A retrospective observational study of Victorian deaths involving MA between 2010 and 2019 was conducted to determine the prevalence and contribution of methylamphetamine (MA) toxicity to death in the absence of other factors. Demographics, autopsy findings, toxicology, and the cause of death were reviewed. Coronial cases were categorized into five groups: deaths due to MA toxicity in the absence of other factors (Group A1); deaths due to MA toxicity in the setting of other potentially contributing factors (Group A2); deaths due to MA toxicity in the setting of significant natural disease (Group B); deaths primarily due to multiple-drug toxicity (Group C); and deaths primarily due to natural causes (Group D). There were 506 deaths involving MA categorized into Group A1 (*n* = 1, 0.6%), Group A2 (*n* = 8, 1.6%), Group B (*n* = 28, 5.5%), Group C (*n* = 229, 45%), and Group D (*n* = 240, 47%). Significant natural disease was prevalent among deaths involving MA and mainly concerned forms of cardiovascular disease (*n* = 277, 55%). The MA concentration in the one death included in Group A1 was 2.1 mg/L. The median MA concentrations of Group A2 (1.6 mg/L) and Group B (0.5 mg/L) were significantly higher than Group C (0.2 mg/L) and Group D (0.2 mg/L). Additionally, many other toxicologically significant drugs were detected and mostly comprised of central nervous system depressants. Deaths due to MA toxicity in the absence of other factors were rare despite the greater availability of crystal MA in the Australian community. The study highlights the interpretative challenges of MA blood concentrations and the continuing harms of this drug in Australia.

**Supplementary Information:**

The online version contains supplementary material available at 10.1007/s12024-023-00724-0.

## Introduction

Methylamphetamine (MA) is a substrate-type releaser that increases the synaptic concentrations of monoamine neurotransmitters [1]. It can be smoked, injected, swallowed, or snorted [2]. At low-to-moderate doses, the effects of MA include arousal, euphoria, positive mood, reduced fatigue, improved attention, behavioral disinhibition, anxiety, suppressed appetite, elevated heart rate and blood pressure, pupil dilation, and increased body temperature [3]. Whereas higher doses of MA may cause acute behavioral disturbances, agitation, motor restlessness, tachycardia, hypertension, chest pain, tachypnoea, dyspnea, hyperpyrexia, rhabdomyolysis, seizures, and multi-organ failure [3]. Recent rises in the global supply of high-purity crystal *S*-( +)-MA have been associated with increases in MA-related harms, including deaths involving MA [4, 5].

Most sudden and unexpected deaths involving amphetamine-type stimulants occur within the context of concomitant drug use [6]. Studies report overlapping blood concentrations between MA-related and MA-associated deaths where tolerance and post-mortem (PM) redistribution were indicated as complicating factors [6–8]. Furthermore, anatomical findings were not characteristic of MA-related deaths, although higher rates of coronary artery disease, myocardial fibrosis, liver portal tract inflammation, and human immunodeficiency viral infection were observed [8]. Darke et al. found deaths attributed to MA toxicity in Australia between 2009 and 2015 were mostly males in their thirties who died after a sudden collapse with high blood concentrations of MA [9]. It is recommended that MA blood concentrations should not be interpreted alone, and the accurate attribution of the cause of death involving MA necessitates a full medico-legal death investigation [7]. For example, a recent review of 104 Japanese autopsy cases from 2014 to 2020 defined accidental intoxications as deaths due to drug toxicity without other fatal findings; only seven accidental mono-intoxications involving MA were reported and the median concentration was 1.2 mg/L [10].

It is hypothesized that most deaths attributed to MA toxicity also implicate natural disease or other drugs despite the greater availability of crystal MA in the Australian community. Thus, we sought to determine the prevalence and contribution of MA toxicity to death in the absence of other factors, specifically significant natural disease and other toxicologically significant drugs.

## Methods

### Data sources

A retrospective observational study of reportable deaths involving MA was conducted using data obtained from the Victorian Institute of Forensic Medicine (VIFM) and the National Coronial Information System (NCIS). Regarding the NCIS, the Victorian Department of Justice and Community Safety was the organizational source of the data and the NCIS was the database source.

The VIFM is a centralized independent statutory authority in Victoria, Australia, that serves a population of ~6.7 million people. Reportable deaths that must be investigated by the Victorian State Coroner include violent, unnatural, and unexpected deaths. The VIFM assists in > 7000 coronial death investigations per year through the provision of forensic medical and scientific services.

As part of the normal process on admission to the mortuary, PM femoral blood was collected by upper thigh arterial puncture as soon as practicable. PM blood specimens were preserved with 1% w/v sodium fluoride and potassium oxalate and stored at 4°C until analysis, usually within 24 h. Ante-mortem (AM) toxicology specimens were obtained where possible if the deceased person died in hospital and stored at −20 °C. The VIFM database was searched for deaths in which MA was detected between 1 January 2010 and 31 December 2019.

The NCIS is a national database on deaths reported to the Coroner in Australia and New Zealand [11]. “Methylamphetamine” was entered into the Query Design search screen and the pharmaceutical substances for human use code set to complement the search of the VIFM database.

### Toxicological analysis

Validated methods of liquid chromatography-tandem mass spectrometry (LC–MS/MS) were used to detect the most common and toxicologically relevant basic and neutral drugs [12]. Toxicologically relevant acidic drugs were detected by an additional LC–MS/MS method [13]. MA blood concentrations were determined by a quantitative LC–MS/MS method. In brief, 100 µL of blood was pipetted into a 2 mL polypropylene tube with added internal standard. Then, 200 µL of trizma base (2 M, pH 9.2) was used to buffer the sample and 1000 µL of butyl chloride was added for a liquid–liquid extraction. The extract was evaporated to dryness and reconstituted in 100 µL of methanol. Samples were injected into the LC–MS/MS system and separated on an Eclipse XBD C18 column (150 × 4.6 mm, 5 µm) using a gradient elution of 50 mM ammonium formate in water (pH 3.5) and 0.1% formic acid in acetonitrile. MA was detected using an Applied Biosystems 5500 Q-TRAP MS/MS (positive electrospray, multiple reaction monitoring mode). The relative standard deviation estimates of accuracy, within- and between-day precision of the method were 3.1, 11, and 16% at 0.03 mg/L; 8.5, 6.6, and 6.6% at 0.45 mg/L; and 2.9, 7.2, and 9.1% at 0.9 mg/L, respectively.

### Medico-legal death investigation in Victoria

Specialist forensic pathologists conduct the medico-legal death investigation in Victoria. A decision is made by the Coroner, with pathologist advice, about the type of autopsy required for each case. Autopsy may be limited to external examination in suitable cases where the cause of death is apparent. Furthermore, external examination alone may also be completed due to objections to internal autopsy by senior next-of-kin based on religious, cultural, or other reasons are considered (these are usually granted). Internal examination of the deceased typically involves investigation of the major organ systems by macroscopy and histology, with additional ancillary tests as required (e.g., vitreous humor, biochemistry, and microbiology). All deceased have a PM computerized tomography scan on admission to VIFM.

### Inclusion and exclusion criteria

The cohort comprised Victorian deaths reported to the Coroner (≥ 18 years old) due to primarily natural causes and accidental drug toxicity in which MA was detected in blood (≥ 0.02 mg/L). Deaths due to other unintentional causes (e.g., motor vehicle crashes or other accidental causes where MA was not included in the cause of death), intentional self-harm, assault, and undetermined causes were excluded. Deceased persons with altered body condition (i.e., traumatized, burnt, or decomposed) identified at mortuary admission were also excluded. Furthermore, cases without internal examination were not included since underlying natural disease may have been an unknown causative or contributory factor to death. Only cases that had PM femoral blood were included and toxicological results of AM blood were preferred if available.

### Case review and classification

Cases were classified into five groups: deaths due to MA toxicity and absence of other factors (Group A1); deaths due to MA toxicity in the setting of other potentially contributing factors (Group A2); deaths due to MA toxicity in the setting of significant natural disease (Group B); deaths primarily due to multiple-drug toxicity (Group C); and deaths primarily due to natural causes (Group D).

Anatomical findings at autopsy were reviewed by a forensic pathologist for significant natural disease defined as any natural disease of the type and severity potentially causal or contributory to death. For example, only severe coronary artery atherosclerosis (≥ 70% stenosis) was defined as significant. Cardiac hypertrophy was determined using an online heart weight calculator (https://calc.chuv.ch/heartweight) and reference cardiac dimensions [14, 15]. Mental illnesses were not considered organic pathologies and were outside the scope of the study.

The contribution of other toxicologically significant drugs was reviewed by a forensic toxicologist. Using similar methods to Pilgrim et al., if one or more drugs were detected at potentially toxic concentrations, the other drugs detected at 'therapeutic' concentrations were considered incidental [6, 16]. For example, in a case with MA and diazepam concentrations of 1 mg/L and 0.2 mg/L, respectively, diazepam was not considered significant. Additionally, MIMS Drug Interactions and CredibleMeds® QTdrugs List were used to assess drug interactions and the risk of QT prolongation, respectively [17, 18]. Drugs administered by medical staff were excluded where medical depositions and ambulance reports permitted. In the absence of 6-acetylmorphine, the likelihood of heroin use relied on the morphine:codeine ratio and circumstantial evidence of intravenous drug use [19].

In some cases, the circumstances suggested the possibility that other factors may have contributed to the death and were classified as Group A2. The final classification of cases was reviewed by a panel of forensic pharmacologists/toxicologists and a forensic pathologist.

### Statistics

Continuous variables were summarized using means or medians, where appropriate, and categorical variables as percentages. Observations with low frequencies have been reported as < 5 unless explicit endorsement was obtained from the Victorian State Coroner. The Kruskal–Wallis *H* test or Mann–Whitney *U* test were used to compare MA concentrations. Assumptions were satisfied and post-hoc analysis was performed by Dunn’s procedure with Bonferroni adjustment for multiple comparisons. *p*-values < 0.05 were considered statistically significant. All statistical analyses were performed using IBM® SPSS® Statistics v28.0.1.1.

### Ethics

This study was approved by the VIFM Ethics Committee (1188–1180/1), Coroners Court of Victoria Research Committee (RC 408), and Justice Human Research Ethics Committee (CF/21/M0481).

## Results

### General

**Fig. 1:**
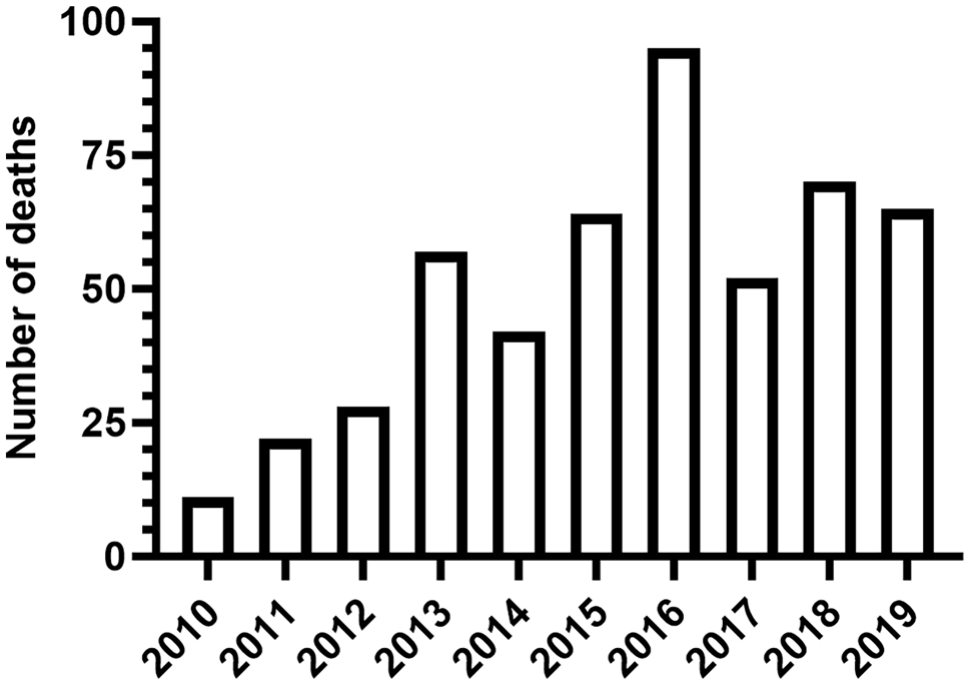
Number of deaths involving MA from 2010 to 2019

There were 1690 reportable deaths where MA was detected in blood (≥ 0.02 mg/L) between 2010 and 2019. Death due to natural causes and accidental drug toxicity comprised 47% of cases (*n* = 787). Other deaths were attributed to other unintentional causes (*n* = 266, 16%), intentional self-harm (*n* = 410, 24%), assault (*n* = 101, 5.9%), or undetermined causes (*n* = 126, 7.5%). The over-representation of MA in motor vehicle crashes and deaths attributed to intentional self-harm has been previously reported [20, 21]. An additional 17% of cases (*n* = 281) were excluded due to body condition altered by trauma, fire, or decomposition (*n* = 42, 2.5%), external examination only (*n* = 214, 13%), altered body condition and central PM blood collection (*n* = 15, 0.89%), or altered body condition and external examination only (*n* = 10, 0.59%). The final cohort included 506 deaths involving MA. Fig. 1 shows how deaths increased over time with the highest prevalence occurring in 2016 (*n* = 95, 19%).

Most deaths were classified into Group C (*n* = 229, 45%) and Group D (*n* = 240, 47%), which was consistent with previous studies where MA was detected but not considered a direct cause of death [6–8]. Indeed, there was only one death in Group A1 (*n* = 1, 0.2%), with a further eight cases in Group A2 (1.6%) where other findings were considered as possible contributing factors but not as definitive as those in the other groups. There were 28 cases in Group B (5.5%) where significant natural disease was recorded by the pathologist. Across all deaths involving MA, the mean age was 42 years old (range = 18–72) and the cohort was largely male (75%).

### Pathology

Significant natural disease was present in 71% (*n* = 358) of all cases and was predominately cardiovascular disease (55%) (refer to Table S1). Additionally, more than one type of pathology was seen in 56% of cases (*n* = 285). The most common pathological entities were cardiac hypertrophy (*n* = 201, 40%), stable ischemic heart disease (*n* = 112, 22%), hypertensive heart disease (*n* = 74, 15%), chronic liver disease (*n* = 56, 11%), pneumonia and infective bronchitis (*n* = 36, 7.1%), unstable ischemic heart disease (*n* = 30, 5.9%), chronic obstructive pulmonary disease (*n* = 20, 4%), infective cardiovascular disease (*n* = 17, 3.4%), asthma (*n* = 16, 3.2%), and hemorrhagic stroke (*n* = 15, 3%). There were 154 obese deceased persons (30%) and 32 were categorized into class III obesity (BMI ≥ 40).

### Toxicology

**Fig. 2:**
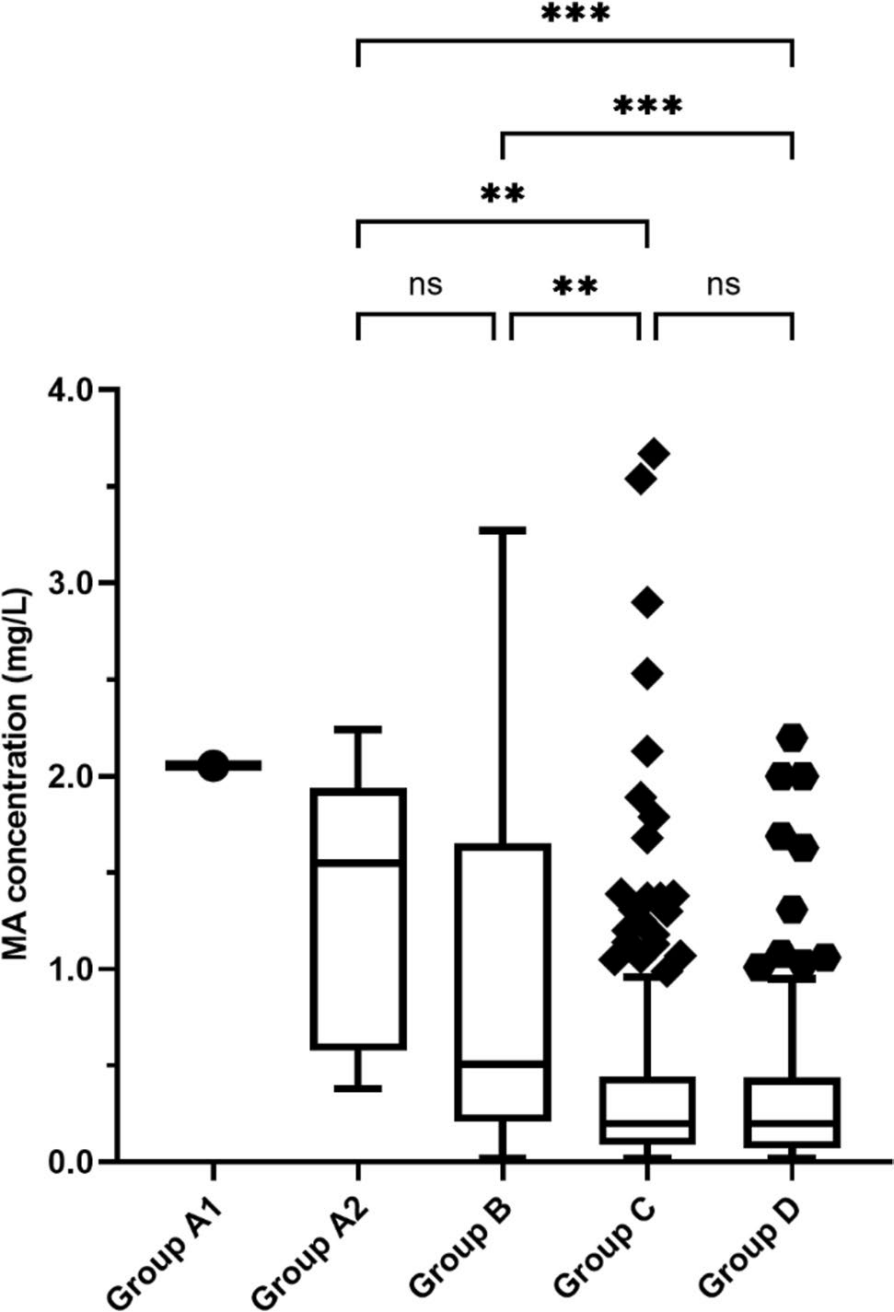
MA concentrations in blood. The *Y*-axis has been truncated at 4.0 mg/L. Outlier values > 4.0 mg/L include the following: 6.0, 6.7, 7.6, and 8.6 (Group B); 7.3 and 23 (Group C). Statistical comparison of the median MA concentration between groups used the Kruskal–Wallis *H* test and Dunn’s procedure using Bonferroni correction for multiple tests (*p < 0.05, **p < 0.01, ***p < 0.001)

MA concentrations are presented in Table 1 and Fig. 2. The median MA and amphetamine concentrations in all deaths involving MA were 0.22 mg/L (IQR = 0.086–0.48, *n* = 506) and 0.067 mg/L (IQR = 0.032–0.11, *n* = 343). The MA concentration in the only Group A1 case was 2.1 mg/L. There was no difference between the median MA concentrations in Group A2 (1.6 mg/L) and Group B (0.5 mg/L) (*p* = 0.690); however, MA concentrations in Group A2 were significantly higher than Group C (0.2 mg/L) (*p* = 0.001) and Group D (0.2 mg/L) (*p* < 0.001). Similarly, MA concentrations in Group B were significantly higher than Group C (*p* = 0.002) and Group D (*p* < 0.001).

**Table 1:** Blood concentrations of MA in 506 deaths involving MA

	Group A1	Group A2	Group B	Group C	Group D
		Median, IQR	Median, IQR	Median, IQR	Median, IQR
*n*	1	8	28	229	240
MA concentration	2.1	1.6, 0.7–1.9^a^	0.5, 0.22–1.6^a^	0.2, 0.088–0.44^b^	0.2, 0.074–0.44^b^
Amphetamine concentration	0.37	0.14, 0.068–0.19^a, b^	0.1, 0.041–0.29^a^	0.062, 0.032–0.011^a, c^	0.049, 0.03–0.083^b, c^
Sum concentration^d^	2.4	1.7, 0.79–2.2^a^	0.64, 0.26–1.9^a^	0.26, 0.11–0.52^b^	0.24, 0.079–0.5^b^
Ratio^e^	0.18	0.11, 0.077–0.14^a^	0.12, 0.088–0.22^a^	0.20, 0.13–0.36^b^	0.17, 0.11–0.29^a, b^

Different letters within rows denote statistically significant differences between respective groups in median values by Dunn’s procedure using Bonferroni correction for multiple tests, excluding Group A1 (refer to Table S2 for Kruskal–Wallis *H* test statistics and the adjusted *p*-values of the Dunn’s procedure pairwise comparison test)

Concentrations are in mg/L

*MA* methylamphetamine, *IQR* inter-quartile range

^d^Sum concentration of MA and amphetamine

^e^Amphetamine:MA concentration ratio

Other toxicologically significant drugs were detected in 56% (*n* = 282) of all deaths involving MA. The most frequently detected drugs included heroin (*n* = 130, 26%), methadone (*n* = 79, 16%), diazepam (*n* = 48, 9.5%), clonazepam (*n* = 29, 5.7%), ethanol (*n* = 23, 4.5%), quetiapine (*n* = 21, 4.2%), tramadol (*n* = 15, 3%), olanzapine (*n* = 13, 2.6%), pregabalin (*n* = 13, 2.6%), and alprazolam (*n* = 12, 2.4%). In addition, delta-9-tetrahydrocannabinol was detected in 27% (*n* = 138) of deceased persons. Again, there was no difference in the MA concentration in the presence or absence of other toxicologically significant drugs, except anti-depressants and opioid narcotics excluding heroin, where it was lower (refer to Table S3).

## Discussion

The increased supply of synthetic stimulants over the past 10 years has been associated with a rise in MA-related harms and has been attributed by some to patterns of consumption of high-purity crystal MA [4, 5]. Deaths in which MA was detected increased between 2010 and 2019, and the majority were categorized into Group C (45% of cases) and Group D (47% of cases). Interestingly, the proportion of deaths categorized into Group D increased over time. It is possible that in an aging population of MA users, the accumulation of natural disease in the setting of chronic MA abuse culminated in more deaths primarily due to natural causes, including those deaths in Group B. In contrast, Table 2 presents the complete case details of the one and eight deaths designated to Group A1 and Group A2, respectively.

**Table 2:** Case details of Group A1 and Group A2 deaths

Case number	Circumstance	Cause of death Autopsy findings	Toxicology	Comment
Group A1				
Case 1	Male in his thirties consumed a "hit" and experienced difficulty breathing. He was later found unconscious and unresponsive. Paramedics attended and pronounced him deceased	*Toxic effects of MA* BMI 25 IVDU stigmata Fibrotic capsule around the left testis	PM femoral blood t1 3.8 h t2 4.7 h MA 2.1 mg/L Amphetamine 0.37 mg/L	Heavy 'ice' user (5 years) and known to smoke/inject MA
Group A2				
Case 2	Male in his forties found deceased at home	*MA toxicity* BMI 34 IVDU stigmata Patchy myocardial fibrosis Hepatosplenomegaly with evidence of chronic hepatitis C Fatty liver Atrophic left testis	PM femoral blood t1 23 h MA 0.93 mg/L Amphetamine 0.11 mg/L Paliperidone 54 ng/mL	Prescribed paliperidone (monthly 150 mg depot injections) and temazepam (not detected) Hx of hepatitis C, necrotising fasciitis, obesity, peripheral neuropathy, schizophrenia, IVDU Illicit drug abuse since the 1980s and MA use continued until the date of his death
Case 3	Male in his thirties returned home and used "ice". He later complained of stomach pains and vomited. He was incoherent with features of cyanosis prior to collapsing the next morning. Paramedics attended and found him in asystole, unresponsive (GCS 3), not breathing, without pulse, and dilated pupils. His body temperature was 34.5 °C. CPR administered by paramedics resulted in ROSC. He developed sinus tachycardia and atrial fibrillation involving weak cardiac output, prior to asystole and was declared dead	*MA toxicity* BMI 29 IVDU stigmata Non-caseating granulomatous inflammation within the lung and the liver Renal impairment Acute hepatitis and hepatitis C Focal cardiac fibrosis	PM femoral blood t1 4.5 h t2 21 h MA 0.46 mg/L Amphetamine 0.076 mg/L Quetiapine 0.045 mg/L	Hx of depression, anxiety, and hepatitis C, IVDU (heroin and MA) Prescribed fluoxetine and quetiapine “heavy” MA user
Case 4	Male in his thirties felt unwell and started hyperventilating due to shortness of breath. He collapsed and became unresponsive. CPR was administered and paramedics attended. He developed asystolic cardiac arrest involving prolonged downtime. CPR administered by paramedics resulted in ROSC. He was intubated and required adrenaline to treat hypotension. In hospital, possible myoclonus was treated with levetiracetam, and he was subsequently sedated with morphine and midazolam. The deceased had no brain activity and developed renal failure. He was pronounced deceased without cardiovascular function five days later	*Hypoxic ischemic encephalopathy secondary to an out of hospital cardiac arrest in the setting of MA use* BMI 26 IVDU stigmata Global cerebral ischemic injury Bronchopneumonia Tracheal ulceration Bilateral pleural effusions Gastric ulceration Right atrial lesion with the characteristics of a papillary fibroelastoma	AM blood t2 16 h MA 1.9 mg/L Amphetamine 0.20 mg/L Hospital drug screen positive for opioids, benzodiazepines, and amphetamines	Hx of IVDU (amphetamines)
Case 5	Male in his fifties smoked MA and was found the next day in the water with heavy equipment including a bag with 30 kg of contents	*MA toxicity in an immersed scuba diver* BMI 29 Mild coronary artery atherosclerosis	PM femoral blood t1 86 h t2 108 h MA 1.9 mg/L Amphetamine 0.38 mg/L	
Case 6	Male in his thirties consuming “ice” and had not slept for three days. He appeared drug affected, displaying features of paranoia and aggression. His behaviour was increasingly erratic and involved strenuous physical activity. The deceased was restrained (without force applied to the back, head, or neck) and became unresponsive without a pulse. CPR was administered and paramedics arrived to provide additional CPR. He was transported to hospital, intubated, and ventilated, however CT brain scan showed widespread hypoxic injuries including oedema, cerebral herniation, and progressive brain swelling, consistent with recent cardiac arrest. Brain death was subsequently pronounced	*Toxicity to MA* BMI 27 Widespread cutaneous abrasions Hypoxic ischemic brain injury Positive myoglobin staining in renal tubules	AM blood MA 0.38 mg/L Amphetamine 0.032 mg/L Hospital drug screen was positive for amphetamines	
Case 7	Male in his twenties reportedly stumbling. He was found in an altered conscious state by paramedics. The man went into cardiac arrest in the ambulance. CPR was commenced. He was hyperthermic (45 °C) with an unrecordable blood pressure. CPR was unsuccessful and he died after continuous cardiac arrests in the emergency department	*Hyperthermia in a man consuming MA and amphetamine* BMI 32 Focal perivascular myocardial fibrosis Mild myocardial vessel dysplasia Mild coronary artery atherosclerosis	PM femoral blood t1 18 h MA 2.2 mg/L Amphetamine 0.16 mg/L Ethanol 0.02 g/100 mL Olanzapine 0.1 mg/L Paliperidone 2 ng/mL Nordiazepam 0.03 mg/L Haloperidol 0.04 mg/L Benztropine 0.1 mg/L	Prescribed clozapine (not detected) Hx of asthma, schizophrenia, and drug abuse (alcohol, cannabis, and MA)
Case 8	Male in his fifties found inside a public toilet between two walls	*Positional asphyxia in the setting of MA use* BMI 27 Multiple abrasions possibly sustained whilst struggling to maneuver between the two walls IVDU stigmata Moderate coronary atherosclerosis Prominent lymphocytic inflammatory infiltrate surrounding the portal tracts Mild hepatic fibrosis	PM femoral blood t1 91 h MA 1.6 mg/L Amphetamine 0.061 mg/L	Hx of amphetamines use since 2011, and MA dependence
Case 9	Male in his thirties was agitated and reportedly experiencing persecutory delusions. He begun destroying property and was confronted by a bystander. There was a physical altercation and the deceased attempted to get into a number of passing vehicles. A second physical altercation occurred which involved an arm around the deceased neck. The man fell to the ground and was retrained (the deceased arms were held behind his back in a prone position, and no force was applied to his back). The deceased was punched multiple times in the head by a second bystander. The deceased reportedly had difficulty breathing, his shirt was damp, skin very hot, and his pulse was “too fast to count”. He became unresponsive and CPR was initiated. He was in cardiac arrest when paramedics arrived with no palpable pulse and fixed dilated pupils. His body temperature was 38.5 °C. CPR was unsuccessful and treatment was ceased	*Cardiorespiratory arrest during prone restraint including pressure on the neck of an obese male using MA* BMI 31 Blunt force trauma to the anterior and posterior neck (intramuscular and soft tissue bruising involving a fracture of the superior horn of the right thyroid cartilage) Mild brain swelling Remote lacunar infarct left caudate head Congested upper chest and head Heavy congested lungs Abrasions over the forehead, nose, left shoulder, and multiple subcutaneous bruises Multiple small and superficial incised injuries to the soles of the feet Linear mucosal laceration on the lower lip Minor left kidney hilar haematoma Mild left ventricular hypertrophy Sigmoid adenomatous polyp Blanching PM lividity over the posterior aspect of the body	PM femoral blood t1 16 h MA 1.5 mg/L Amphetamine 0.18 mg/L	Hx of depression, post-traumatic stress disorder, and drug abuse (MA)

*AM* ante-mortem, *BMI* body mass index, *CPR* cardiopulmonary resuscitation, *CT* computed tomography, *GCS* Glasgow Coma Scale, *Hx* history, *IVDU* intravenous drug user, *MA* methylamphetamine, *PM* post-mortem, *ROSC* return of spontaneous circulation, *t1* time (h) between death and PM blood time of collection (TOC), *t2* survival time (h) between last known MA dose and specimen TOC

The acute toxicities of amphetamines previously described by Ellinwood and Lee include the following: cardiotoxicities associated with a sympathetic over-stimulation, cardiovascular catastrophe caused by a surge in blood pressure on a background of vascular abnormalities, amphetamines-induced hyperthermia, which can lead to rhabdomyolysis, and seizures and associated anoxia due to a lowered seizure threshold [22].

A review of the cases presented in Table 2 revealed other factors that may have increased the risk of death, thus cannot be definitely categorized as deaths due to the direct toxic effects of MA (i.e., circumstances, autopsy findings, or toxicology were present that introduce reasonable doubt about the sole causal role of MA toxicity). For example, drowning or narcosis in case 5 and positional asphyxia in case 8 are plausible competing causes of death. Furthermore, the acute behavioral disturbance and hyperadrenergic emotional state in the setting of physical restraint in cases 6 and 9 cannot be ignored as contributors to death [23]. Matsumoto et al. contend that the hyperthermic actions of MA, in general, involve exacerbating factors of body temperature dysregulation and develop over many hours [24]. The condition of the deceased in case 7 rapidly deteriorated and he was pronounced deceased within 70 min of first contact with paramedics. There is limited information about the events prior to the arrival of paramedics on the scene, although the ambient temperature was in the high thirties. In cases 3 and 4, symptoms of probable MA toxicity prior to unresponsiveness and cardiac arrhythmia were reported, although the contribution of other drugs could not be entirely excluded despite their therapeutic indication and/or low concentration. In a similar way, paliperidone is known to prolong the QT-interval and drug interactions may have contributed to death in case 2 [18]. Case 1 was the only death among the cohort due to the direct toxic effects of MA. Again, difficulty breathing and unresponsiveness may have preceded a sudden cardiac event. It is also noteworthy that no cardiovascular disease was found in case 1 despite the deceased’s five-year history of “heavy” MA abuse. Lethal cardiac arrhythmias in the absence of cardiac structural abnormalities does not preclude the possibility of an underlying cardiac channelopathy, and long QT syndrome and catecholaminergic polymorphic ventricular tachycardia may be triggered by adrenergic stimuli; however, none of these conditions were suspected in these cases [25].

Thorough internal examination is essential due to the high prevalence of co-morbidities and the well-established link between MA abuse and cardiovascular pathology [6–8]. Defining a minimum concentration of MA sufficient to cause specific mechanisms of toxicity is not possible and is confounded by the effects of tolerance and PM redistribution [26, 27]. Furthermore, there was no difference in blood concentrations between Group A2 and Group B; therefore, it is not unreasonable to conclude that the high MA concentrations in Group B may have caused death in the absence of significant natural disease.

The involvement of other toxicologically significant drugs and possible drug interactions that intensify drug toxicity may explain the lower MA concentrations in Group C. Most other toxicologically significant drugs detected in these cases have central nervous system and respiratory depressant effects, for example, heroin and methadone. The high prevalence of MA in heroin-related deaths has been previously reported in the United States as the conflation of “twin epidemics,” and reinforces the dangers of combining these drugs which contradicts the lay perception among opioid users that MA provides an accessible harm reduction strategy [28, 29].

### Limitations

A few limitations to this study need to be acknowledged. Firstly, the single center retrospective design may lack generalizability, and the rarity of deaths due to MA toxicity in the absence of other factors may be peculiar to the studied population in Victoria. That said, Karch suggested the same in 1999 [30]. Discretionary toxicological testing for acidic drugs, gamma-hydroxybutyrate, and some novel psychoactive substances may have missed positive cases. The exclusion of cases (*n* = 281) due to abnormal body condition, collection of central PM blood, or external examination only may have omitted relevant deaths involving MA. Cautious interpretation of the blood concentrations of MA in Group A1 and Group A2 is needed due to the very limited number of cases. Furthermore, future studies designed to test hypotheses of an accelerated rate of age-related natural disease in MA-related deaths would benefit from the inclusion of a suitable control group to permit an adjustment for background incidence.

## Conclusion

This retrospective observational study of 506 deaths involving MA over 10 years demonstrates that deaths due to MA toxicity in the absence of other factors are exceptionally rare. Deaths primarily due to natural causes were highly prevalent, especially cardiovascular-related disease; therefore, internal examination as a component of the medico-legal death investigation is essential to determine the most reasonable cause of death in the absence of obvious external causes. Comprehensive toxicological analysis is also highly recommended in suspected MA-associated deaths due to the prevalence of other toxicologically relevant drugs. The complexity of these deaths highlights the difficulty in ascribing the significance of MA blood concentrations and the continuing harms associated with MA in Australia.

## Key points

1. Deaths in which MA was detected increased over time.
2. Most deaths involved significant natural disease and other toxicologically significant drugs.
3. Deaths due to the direct toxic effects of MA in the absence of other factors were rare.

## Supplementary Information

Below is the link to the electronic supplementary material.

Supplementary file1 (DOCX 41 KB)
