# Supplementary material for: Methylamphetamine toxicity and its involvement in death: A retrospective observational study of deaths reported to the Victorian Coroner, Australia
Source: Forensic Sci Med Pathol. 2023 Oct 4;20(3):852–62. doi: 10.1007/s12024-023-00724-0 (PMC11525310; doi:10.1007/s12024-023-00724-0)
Supplement: Supplementary file 1 — Supplementary file1 (DOCX 41 KB) [file 12024_2023_724_MOESM1_ESM.docx]

**Table S1** Cohort characteristics and pathology of deaths involving methylamphetamine (MA)

|  | All deaths involving MA | Group A2 | Group B | Group C | Group D |
| --- | --- | --- | --- | --- | --- |
| Case details |  |  |  |  |  |
| Sample number (n, %) | 506, 100 | 8, 1.6 | 28, 5.5 | 229, 45 | 240, 47 |
| Age, years (mean, range) | 42, 18–72 | 41, 24–53 | 41, 21–65 | 38, 19–67 | 45, 18–72 |
| Gender, male (n, %) | 379, 75 | 8, 100 | 24, 86 | 153, 67 | 193, 80 |
| BMI (mean, range) | 27, 13–64 | 29, 26–34 | 28, 19–42 | 27, 13–64 | 28, 14–58 |
| Post-mortem interval, h (median, IQR) | 8.8, 4.4–18 | 17, 2.3–54 | 7.1, 0–16 | 9.8, 5.3–19 | 7.4, 3.9–16 |
| Pathology (n, %) |  |  |  |  |  |
| Any significant natural disease | 358, 71 |  | 28, 100 | 90, 39 | 240, 100 |
| Any significant central nervous system disease | 30, 5.9 |  | < 5 | < 5 | 26, 11 |
| Hemorrhagic stroke | 15, 3 |  |  |  | 15, 6.3 |
| Ischemic stroke |  |  |  |  |  |
| Epilepsy and other seizure disorders | 10, 2 |  |  | < 5 | 7, 2.9 |
| Any significant cardiovascular disease | 277, 55 |  | 21, 75 | 67, 29 | 189, 79 |
| Stable ischemic heart disease* | 112, 22 |  | 7, 25 | 21, 9.2 | 84, 35 |
| Unstable ischemic heart disease† | 30, 5.9 |  |  | < 5 | 29, 12 |
| Hypertensive heart disease | 74, 15 |  | < 5 | 25, 11 | 45, 19 |
| Cardiomyopathy | 11, 2.2 |  | < 5 | < 5 | 9, 3.8 |
| Cardiac hypertrophy | 201, 40 |  | 17, 61 | 54, 24 | 130, 54 |
| Valvular disease | < 5 |  |  |  | < 5 |
| Aortic and other artery dissection | 6, 1.2 |  |  |  | 6, 2.5 |
| Pulmonary thromboembolism | 11, 2.2 |  |  |  | 11, 4.6 |
| Infective cardiovascular disease | 17, 3.4 |  | < 5 |  | 16, 6.7 |
| Congenital heart disease | 6, 1.2 |  |  | < 5 | 5, 2.1 |
| Any significant respiratory disease | 71, 14 |  | < 5 | 16, 7 | 52, 22 |
| Asthma | 16, 3.2 |  | < 5 | < 5 | 13, 5.4 |
| Pneumonia and infective bronchitis‡ | 36, 7.1 |  |  | 11, 4.8 | 25, 10 |
| Emphysema and/or chronic bronchitis | 20, 4 |  | < 5 | 5, 2.2 | 13, 5.4 |
| Any significant gastrointestinal disease | 65, 13 |  | 5, 18 | 24, 11 | 36, 15 |
| Chronic liver disease (cirrhosis and severe steatosis) | 56, 11 |  | 5, 18 | 24, 11 | 27, 11 |
| Any significant renal disease | 8, 1.6 |  | < 5 | < 5 | 6, 2.5 |
| Chronic kidney disease§ | < 5 |  | < 5 |  | < 5 |
| Any significant endocrine disease | 17, 3.4 |  | < 5 |  | 15, 6.3 |
| Uncontrolled diabetes mellitus¶ | 12, 2.4 |  |  |  | 12, 5 |
| Stable diabetes mellitus | < 5 |  | < 5 |  | < 5 |
| Sepsis | 14, 2.8 |  | < 5 | < 5 | 12, 5 |
| Other significant natural disease including cancer** | 21, 4.2 |  | < 5 | < 5 | 19, 7.9 |

BMI, body mass index; IQR, inter-quartile range; MA, methylamphetamine

Percentage values represent proportions within cause of death groups (Group A1 not presented, refer to Table 2)

* Severe coronary artery stenosis (> 70%) with or without myocardial fibrosis, coronary artery stent in suti, or coronary artery bypass graft

† Coronary artery thrombus, acute myocardial infarction, or contraction band necrosis

‡ Viral pneumonia, bronchopneumonia (non-agonal), or aspiration pneumonia (non-agonal)

§ Glomerulosclerosis, nephrosclerosis, hypertensive renal damage, severe diabetic nephropathy, or renal failure

¶ Diabetic ketoacidosis and hyperosmolar hyperglycemic state

** Cerebral artery vasculitis, meningitis, spinal muscular atrophy, acquired brain injury, congestive cardiac failure, ruptured aortic and other artery aneurysm, non-coronary artery and non-pulmonary artery thrombosis, cardiac pacemaker and other past cardiac surgery, obstructive sleep apnea, pulmonary fibrosis, retropharyngeal abscess, upper gastrointestinal hemorrhage, pancreatitis, peritonitis, ischemic bowel, inflammatory esophageal pathology, hemoperitoneum, hydronephrosis, pyelonephritis, malignant hypertension and cancer

**Table S2** Kruskal-Wallis H test statistics and Dunn’s procedure pairwise comparison test statistics

|  | Kruskal-Wallis H test statistics | | | Dunn’s procedure test statistics | |
| --- | --- | --- | --- | --- | --- |
|  | df | Χ2 | *p* value | t | *p* value* |
| MA concentration | 3 | 31.251 | < .001 |  |  |
| Group A2 v Group B |  |  |  | 1.576 | .690 |
| Group A2 v Group C |  |  |  | 3.764 | .001 |
| Group A2 v Group D |  |  |  | 4.029 | < .001 |
| Group B v Group C |  |  |  | 3.607 | .002 |
| Group B v Group D |  |  |  | 4.087 | < .001 |
| Group C v Group D |  |  |  | 1.020 | 1.000 |
| Amphetamine concentration | 3 | 13.276 | .004 |  |  |
| Group A2 v Group B |  |  |  | .725 | 1.000 |
| Group A2 v Group C |  |  |  | 1.871 | .368 |
| Group A2 v Group D |  |  |  | 2.440 | .088 |
| Group B v Group C |  |  |  | 1.818 | .415 |
| Group B v Group D |  |  |  | 2.788 | .032 |
| Group C v Group D |  |  |  | 1.818 | .415 |
| Sum concentration | 3 | 31.756 | < .001 |  |  |
| Group A2 v Group B |  |  |  | 1.538 | 0.744 |
| Group A2 v Group C |  |  |  | 3.700 | .001 |
| Group A2 v Group D |  |  |  | 4.030 | < .001 |
| Group B v Group C |  |  |  | 3.566 | .002 |
| Group B v Group D |  |  |  | 4.165 | < .001 |
| Group C v Group D |  |  |  | 1.275 | 1.000 |
| Ratio^†^ | 3 | 15.664 | .001 |  |  |
| Group A2 v Group B |  |  |  | -.905 | 1.000 |
| Group A2 v Group C |  |  |  | -2.760 | .035 |
| Group A2 v Group D |  |  |  | -2.228 | .155 |
| Group B v Group C |  |  |  | -2.995 | .016 |
| Group B v Group D |  |  |  | -2.083 | .223 |
| Group C v Group D |  |  |  | 1.684 | .553 |

MA, methylamphetamine

* adjusted *p* values of Bonferroni correction for multiple tests

† amphetamine:MA concentration ratio

**Table S3** MA concentrations (mg/L) in the presence or absence of other toxicologically significant drugs

|  |  |  | Toxicologically  significant  drug present | Toxicologically  significant  drug absent | Mann-Whitney U test statistics | | |
| --- | --- | --- | --- | --- | --- | --- | --- |
|  | n | % | Median, IQR | Median, IQR | U | z | *p* value§§ |
| Ethanol | 23 | 4.5 | 0.13, 0.049–0.26 | 0.23, 0.088–0.49 | 4143.500 | -2.060 | .039 |
| Other amphetamine-type stimulants* | 6 | 1.2 | 0.25, 0.074–0.55 | 0.22, 0.087–0.47 | 1500.000 | 0.000 | 1.000 |
| Anti-convulsants† | 14 | 2.8 | 0.26, 0.084–0.64 | 0.22, 0.087–0.47 | 3526.500 | 0.153 | .878 |
| Anti-depressants‡ | 41 | 8.1 | 0.14, 0.054–0.33 | 0.24, 0.090–0.49 | 7424.500 | -2.349 | .019 |
| Anti-psychotics§ | 40 | 7.9 | 0.19, 0.076–0.33 | 0.23, 0.088–0.49 | 7908.500 | -1.591 | .112 |
| Benzodiazepines¶ | 86 | 17 | 0.20, 0.079–0.42 | 0.23, 0.089–0.49 | 16658.500 | -1.135 | .257 |
| Cannabis** | 138 | 27 | 0.19, 0.071–0.43 | 0.25, 0.088–0.50 | 23242.500 | -1.467 | .142 |
| Cocaine | 8 | 1.6 | 0.31, 0.13–1.1 | 0.22, 0.085–0.47 | 2446.500 | 1.108 | .268 |
| Heroin | 130 | 26 | 0.26, 0.11–0.49 | 0.21, 0.082–0.47 | 26032.000 | 1.108 | .268 |
| Novel psychoactive substances | < 5 |  | 0.11, 0.048–0.42 | 0.22, 0.088–0.48 | 764.000 | -0.824 | .410 |
| Opioid narcotics†† | 121 | 24 | 0.17, 0.065–0.36 | 0.25, 0.10–0.50 | 19177.000 | -2.934 | .003 |
| Gamma-hydroxybutyrate | 9 | 1.8 | 0.26, 0.058–2.9 | 0.22, 0.088–0.47 | 2505.500 | 0.619 | .536 |
| Other substances classed as drugs‡‡ | 16 | 3.2 | 0.24, 0.061–0.39 | 0.22, 0.088–0.48 | 3846.000 | -0.129 | .898 |

MA, methylamphetamine

* 3,4-methylenedioxymethylamphetamine and 3,4-methylenedioxyamphetamine

† Gabapentin and pregabalin

‡ Amitriptyline, citalopram, clomipramine, desvenlafaxine, doxepin, duloxetine, fluoxetine, mirtazapine, nortriptyline, paroxetine, sertraline and venlafaxine

§ Amisulpride, aripiprazole, chlorpromazine, clozapine, flupentixol, olanzapine, paliperidone, quetiapine, risperidone and zuclopenthixol

¶ Alprazolam, clonazepam and 7-aminoclonazepam, diazepam and nordiazepam, etizolam, lorazepam, nitrazepam and 7-aminonitrazepam, oxazepam, temazepam, zolpidem and zopiclone

** Cannabis not considered toxicologically significant and represents delta-9-tetrahydrocannabinol detected or not detected

†† Codeine, buprenorphine and norbuprenorphine, diphenhydramine, fentanyl, hydromorphone, methadone, morphine, oxycodone, pholcodine and tramadol

‡‡ Anesthetics, analgesics, antibiotics, anti-diabetics, anti-histamines, anti-inflammatories, asthma, barbiturates, carbon monoxide, cardiovascular drugs, diuretics, endogenous substances, muscle relaxants and anti-spastics, stimulants and anorectics, volatile substances, and other substances classed as drugs

§§ Bonferroni-corrected *p* value = .0038
